# Rapid effectiveness of eptinezumab to treat ongoing migraine attacks: preliminary data from an Italian multicenter real-world experience (the BE-FREE study)

**DOI:** 10.3389/fneur.2026.1784242

**Published:** 2026-03-31

**Authors:** Luisa Fofi, Claudia Altamura, Marilena Marcosano, Alberto Doretti, Francesca Pistoia, Antonio Granato, Simona Guerzoni, Michele Trimboli, Licia Grazzi, Danilo Antonio Montisano, Gianluca Avino, Gabriele Sebastianelli, Carla Fasano, Elisa Maria Piella, Pierangelo Geppetti, Nicoletta Brunelli, Luigi Francesco Iannone, Fabrizio Vernieri

**Affiliations:** 1 Neurosonology and Headache Unit, Fondazione Policlinico Universitario Campus Bio-Medico, Rome, Italy; 2 Department of Medicine and Surgery, Università Campus Bio-Medico di Roma, Rome, Italy; 3 Department of Neurology-Laboratory of Neuroscience, IRCCS, Istituto Auxologico Italiano, Milan, Italy; 4 Department of Biotechnological and Applied Clinical Sciences, Neurological Institute, University of L’Aquila, L’Aquila, Italy; 5 Headache Center, Neurology Clinic, University Hospital of Trieste, University of Trieste, Trieste, Italy; 6 Digital and Predictive Medicine, Pharmacology and Clinical Metabolic Toxicology-Headache Center and Drug Abuse-Laboratory of Clinical Pharmacology and Pharmacogenomics, Department of Specialist Medicines, AOU Policlinico di Modena, Modena, Italy; 7 Azienda Ospedaliera-Universitaria “Renato Dulbecco”, Catanzaro, Italy; 8 Headache Center, Department of Neurology, IRCCS Foundation “Carlo Besta” Neurological Institute, Milan, Italy; 9 SOC Neurologia, Ospedale Santo Stefano, USL Toscana Centro, Prato, Italy; 10 Department of Medico-Surgical Sciences and Biotechnologies, Polo Pontino ICOT, Sapienza University of Rome, Latina, Italy; 11 Headache and Cerebrovascular Diseases Center, Neurology Unit 2, Careggi University Hospital, Florence, Italy; 12 Department of Neurosciences “Rita Levi Montalcini”, University of Turin, Turin, Italy; 13 Department of Molecular Pathobiology and Pain Research Center, College of Dentistry, New York University, New York, NY, United States; 14 Department of Biomedical, Metabolic and Neural Sciences, University of Modena and Reggio Emilia, Modena, Italy

**Keywords:** migraine, migraine attack, eptinezumab, acute treatment, effectiveness

## Abstract

**Introduction:**

Eptinezumab is an intravenous anti-calcitonin gene-related peptide (CGRP) monoclonal antibody (mAb) approved for the prevention of episodic and chronic migraine. We aimed to explore the earliest changes in migraine pain intensity and associated symptoms during the first 30 min of eptinezumab infusion in a real-world setting, particularly when an acute migraine attack was ongoing.

**Methods:**

The BE-FREE study is an ongoing Italian observational, multicenter, independent, real-world, and prospective study. We enrolled patients affected with episodic migraine (EM) and chronic migraine (CM) who were experiencing an ongoing migraine attack. Eptinezumab was administered within 1–12 h of the onset of the qualifying migraine attack. Data collected included monthly migraine days (MMDs), headache pain intensity, the number of monthly acute medications, and the use of concomitant or past standard preventive treatments (SPTs). The Numerical Rating Scale (NRS), associated symptoms, and pain freedom (PF) were recorded before infusion (T0) and at intervals of 10 (T10), 20 (T20), and 30 (T30) min during infusion.

**Results:**

We enrolled 31 patients (87% female), with a mean age of 43.1 years (SD 2.7); of these, 68% had CM. NRS scores significantly reduced at T10 (*p* = 0.011) and T20 (*p* = 0.004). Photophobia was less frequent at T10 (*p* = 0.045), phonophobia at T20 (*p* = 0.014), and osmophobia at T30 (*p* = 0.046) compared with T0. PF was reported by 6.5% of patients at T10, 19.4% at T20, 29.0% at T30, and 19.4% at T60, T90, and T120.

**Discussion:**

The BE-FREE study results, representing the first real-world evidence, demonstrate the rapid effect of eptinezumab during the first 30 min of infusion in patients experiencing an ongoing migraine attack.

## Introduction

Eptinezumab is a humanized intravenous anti-calcitonin gene-related peptide (CGRP) monoclonal antibody (mAb) approved in 2022 in Europe for the preventive treatment of migraine in adults who have at least 4 monthly migraine days (MMDs) (1). Eptinezumab is administered as a 30-min infusion every 12 weeks at a dose of 100 mg or 300 mg (2), and it is the only antibody targeting the CGRP ligand that is administered intravenously.

After intravenous administration, eptinezumab reaches its maximal serum concentration (*C*_max_) at the end of the infusion, with 100% bioavailability. Compared with other subcutaneous anti-CGRP mAbs, eptinezumab demonstrates a shorter maximal serum concentration (*C*_max_) and greater exposure following administration (3, 4).

The immediate and sustained effect of eptinezumab can be explained by its binding properties. Eptinezumab binds CGRP potently and rapidly, undergoing conformational changes in the fragment antigen-binding (Fab) region through a specific “latch-and-lock” mechanism, which results in slow dissociation from the peptide and explains both rapid onset and sustained preventive effect in migraine (5).

Randomized clinical trials [PROMISE-1 (6), PROMISE-2 (7), RELIEF (8, 9), and DELIVER (10)] and the open-label [PREVAIL (11)] study conducted in patients with both episodic migraine (EM) and chronic migraine (CM) demonstrated a significant positive effect of eptinezumab compared with placebo in alleviating migraine frequency, reducing the mean MMDs, increasing the MMD responder rates (≥50% and ≥75%), and improving patient-reported outcome measures.

The potential effect of eptinezumab on the acute phase of a migraine attack was studied in the RELIEF trial (8, 9), a phase 3, multicenter, parallel-group, double-blind, placebo-controlled clinical trial in which patients affected by episodic migraine were randomized to receive 100 mg of eptinezumab or placebo intravenously during a moderate-to-severe migraine attack. Patients (241 in the eptinezumab group vs. 244 in the placebo group) were evaluated at different time points after the infusion (0.5, 1, 1.5, 2, 2.5, 3, 3.5, 4, 6, 9, and 12 h) and at 24 and 48 h. In the first report, 23.5% of patients treated with eptinezumab achieved pain freedom within 2 h (compared with 4 h in the placebo group; *p* < 0.001), and 55.5% of patients achieved absence of the most bothersome symptoms within 2 h (compared with 3 h in the placebo group; *p* < 0.001). In a subsequent exploratory analysis (9), the authors described higher rates of absence of photophobia (29.4% vs. 17.0%) and absence of phonophobia (41.2% vs. 27.2%) at 1 h and during the following 48 h (*p* < 0.05) in the group treated with eptinezumab compared with the placebo group, respectively.

In this study, for the first time, we explored the effectiveness of eptinezumab in a real-world setting on the change in migraine pain intensity and associated symptoms during an acute migraine attack, focusing on the first 30 min of eptinezumab infusion.

## Materials and methods

The BE-FREE is an ongoing Italian observational, multicenter, investigator-initiated, independent, real-world, prospective study conducted as a sub-study of the Italian Headache Registry [*Registro Italiano delle Cefalee (RICe)*] promoted by the Italian Society for the Study of Headaches (SISC) and approved by the Ethics Committee of the AOU Careggi, Florence, on 20 March 2019 (CEAVC Studio RICe, 14591_oss and subsequent amendments). Details about the RICe are reported elsewhere (12). All patients provided written informed consent.

We considered all consecutive adult outpatients treated with eptinezumab (100 mg or 300 mg) for EM and CM according to the International Classification of Headache Disorders (ICHD-3) (13) criteria from November 2024 to May 2025.

We evaluated patients scheduled for a planned eptinezumab infusion (100 mg or 300 mg, according to clinical practice) at participating Italian headache centers, regardless of the dose and cycle of infusion, and enrolled in our study only those experiencing an ongoing acute migraine attack. Patients who had taken acute medications within the 24 h preceding the infusion or during the infusion were excluded.

Before the eptinezumab infusion, baseline migraine features—including MMDs, headache pain intensity (measured by NRS 0–10), the number of monthly acute medications (MAMs), use of standard preventive treatments (SPTs, i.e., antiepileptics, antidepressants, beta-blockers, and calcium antagonists), past SPT failures, spontaneous remission of migraine attacks in the past 5 years, and medication overuse headache (MOH)—were collected.

Furthermore, headache pain intensity measured by the NRS score, the presence of accompanying symptoms (i.e., photophobia, phonophobia, osmophobia, nausea, and vomiting), and acute medication use were recorded at baseline [i.e., before the infusion (T0)] and at 10 min (T10), 20 min (T20), 30 min (T30), 1 h (T60), 1.5 h (T90), and 2 h (T120) after the infusion started.

### Outcomes

The primary outcome was the earliest significant change in NRS score at different time points during and after the start of the infusion.

The secondary outcomes were as follows: (i) to assess the earliest significant change in the frequency of accompanying symptoms compared with baseline and (ii) to achieve pain freedom at different time points after the infusion started.

### Statistical analysis

The present study is a preliminary, *a priori* analysis of a convenience sample of patients. Data distribution was assessed for normality using the Kolmogorov–Smirnov test. Variables were reported as means with standard deviations (SD) or medians with interquartile range (IQR), according to data distribution. We used the Wilcoxon signed-rank test to analyze the changes of interval variables without normal distribution. The McNemar test for proportions of paired samples was used to assess changes in the frequency of categorical variables over the evaluation times. Contingency tables (chi-squared tests) and unadjusted odds ratios (ORs) with their 95% confidence intervals (CIs) were used to compare frequencies between groups. All tests were two-tailed. Statistical significance was set at a two-tailed *p*-value of <0.05. We included only individuals with complete information regarding the primary studied variable (NRS). We declared data availability and ran the analysis only in patients with usable data for the secondary variables. Statistical analyses were performed using SPSS version 27.0 (SPSS Inc., Chicago, IL, United States).

## Results

**Figure 1:**
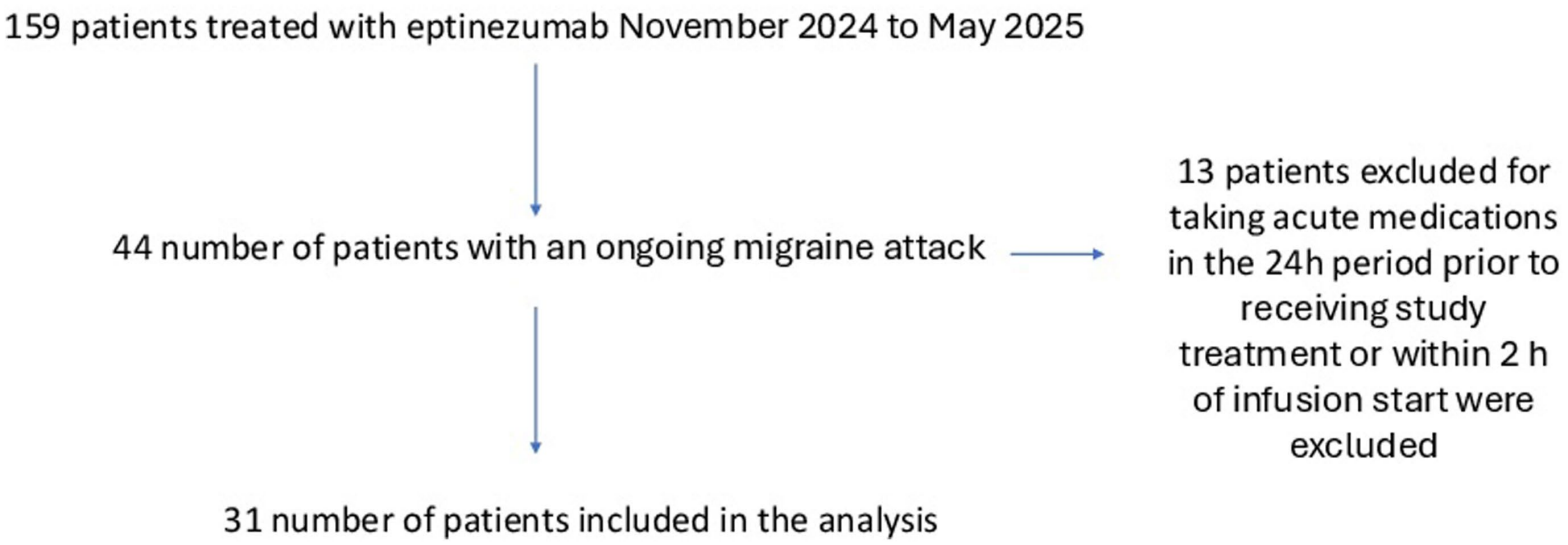
Flowchart.

We enrolled 31 patients (see Figure 1), of whom 27 were female (87%), with a mean age of 43.1 (SD 2.7) years. Eptinezumab was administered at a dose of 100 mg to 19 patients (61.2%) and at a dose of 300 mg to 12 patients (38.8%).

Data were collected at different cycles of eptinezumab infusions: I infusion (16 patients, 51.6%), II (7 patients, 22.6%), III (3 patients, 9.7%), and IV (5 patients, 16.1%). Before the eptinezumab infusion, MMDs were 23.0 (IQR: 15), 68% of patients had CM, 62% had MOH, and 35% of patients had ongoing stable SPTs for at least 3 months.

At T0, patients reported that the attack had started 3.9 ± 2.4 h before the infusion; none reported a spontaneous remission of their usual migraine attacks in their past 5 years.

**Figure 2:**
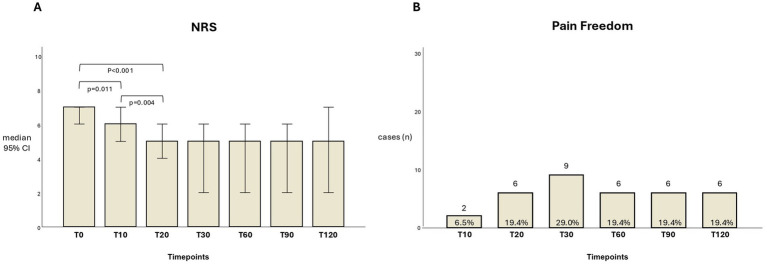
**(A)** The bars indicate the NRS median value (range: 0–10, confidence interval: 95%) along the different evaluation times. **(B)** The bars indicate the number of patients achieving pain freedom at different evaluation times; the percentages out of the cohort are also reported.

The NRS score significantly decreased from T0 (7.0, IQR: 2) to T10 (6.0, IQR: 3; *p* = 0.011) and from T10 to T20 (5.0, IQR: 3; *p* = 0.004), with a significant trend also from T20 to T30 (5.0, IQR: 5; *p* = 0.056). No significant changes occurred at the following observed time points, as shown in Figure 2A. Pain freedom (PF) was reported by 6.5% of patients at T10, 19.4% at T20, 29.0% at T30, and 19.4% at T60, T90, and T120 (Figure 2B).

**Figure 3:**
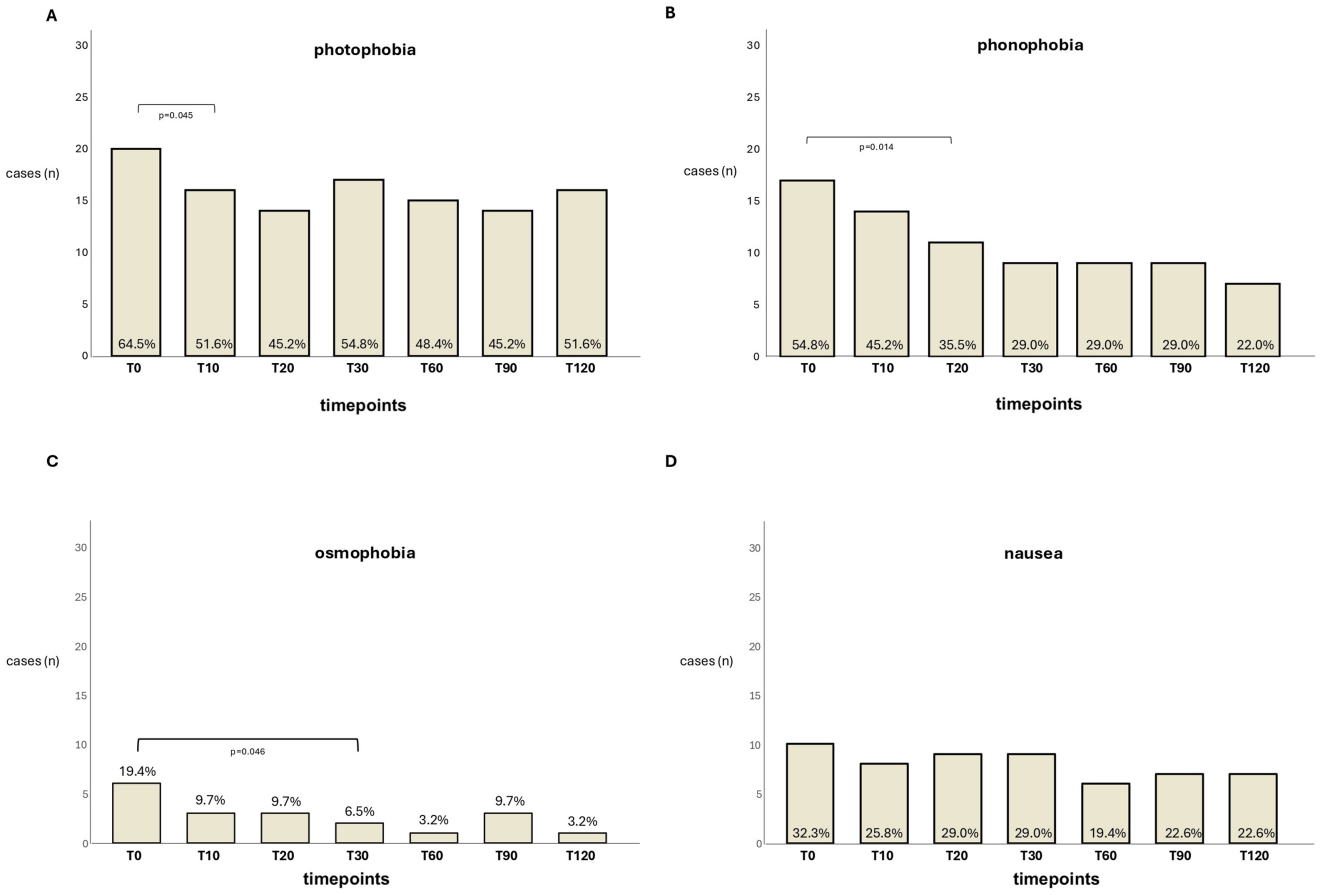
The bars indicate the number of patients reporting associated symptoms at the different evaluation times: **(A)** photophobia, **(B)** phonophobia, **(C)** osmophobia, and **(D)** nausea. The percentages out of the cohort are also reported.

Regarding accompanying symptoms, the presence of photophobia was less frequent at T10 (*p* = 0.045) compared with baseline; no further significant changes were observed at the subsequent time points (Figure 3A). Compared with the baseline, the earliest reduction in the frequency of phonophobia was observed at T20 (*p* = 0.014, Figure 3B) and of osmophobia at T30 (*p* = 0.046, Figure 3C). The frequency of nausea did not change significantly at any of the intervals (*p* > 0.05, Figure 3D).

Different doses of eptinezumab (100 mg vs. 300 mg) and the presence of CM did not influence the remission of associated symptoms or pain freedom at the different time points (consistently *p* > 0.10).

## Discussion

Current evidence demonstrates that eptinezumab exerts a potent and sustained migraine preventive effect in patients with EM and CM (6, 7, 10, 11). Our study revealed that eptinezumab also has a fast-acting action, reducing migraine pain intensity and associated symptoms as early as 10 min after the start of the infusion in patients with ongoing migraine attacks.

Pain intensity and accompanying symptoms significantly improved within the first 30 min, except for nausea. Moreover, pain freedom was achieved in approximately 30% of patients within the first 30 min after the infusion (including 6.5% within 10 min) and in approximately 20% of patients at 2 h.

These findings support the hypothesis that eptinezumab is fast-acting, even during the 30-min infusion, before reaching *T*_max_ (1 h), as observed in the RELIEF study (8, 9).

The clinical improvement observed in our study and in the RELIEF trial after the first 10 min of the infusion suggests that eptinezumab acts within minutes, most likely on peripheral components of migraine pathophysiology (i.e., trigeminovascular) (14, 15).

In the RELIEF study, patients with EM (7.2 ± 2.7 MMDs at baseline) received 100 mg of eptinezumab (*n* = 238) and placebo (*n* = 242) during a migraine attack. In the first 30 min following the start of infusion, pain freedom and headache relief did not differ significantly between the study groups; only the absence of MBS varied significantly (19.3% vs. 22.1%, *p* = 0.067). After 1 h, patients treated with eptinezumab achieved more significant headache pain freedom (9.7% vs. 4.1%, *p* = 0.001), pain relief (38.7% vs. 26.9%, *p* = 0.005), and absence of most bothersome symptoms (MBS) (33.2% vs. 22.1%, *p* = 0065) than those receiving placebo.

In our study, significant changes in pain intensity and accompanying symptoms were observed within the first 30 min after the infusion. A significant reduction in photophobia frequency occurred as early as T10 (*p* = 0.025), phonophobia at T20 (*p* = 0.005), and osmophobia at T30 (*p* = 0.046) compared with baseline.

Although the BE-FREE study is based on a small sample, it confirms that eptinezumab initiates its anti-migraine action from the very first stages after infusion, including in patients with CM (68%) and MOH (62%), who are more severely impaired than those enrolled in the RELIEF study.

In this preliminary analysis, we decided not to separate data of patients at their first infusion from those at subsequent infusions due to the small sample size. It should be noted that a residual amount of the drug from previous administrations could still be present in the blood.

We cannot exclude a placebo response with parenteral treatment, even though the majority of patients were well known at the headache center and had been previously treated with multiple therapies, and the changes described were directly observed by the neurologists.

The BE-FREE is the first study to report the rapid effect of eptinezumab in treating ongoing migraine attacks during the first 30 min of the infusion in a real-world setting.

## Data Availability

The datasets presented in this study can be found in online repositories. The names of the repository/repositories and accession number(s) can be found in the article/supplementary material.

## Glossary

CGRP: Calcitonin gene-related peptide
EM: Episodic migraine
CM: Chronic migraine
SPT: Standard preventive treatment
PF: Headache pain freedom
T0: Baseline before infusion
T10: 10 min after the infusion start
T20: 20 min after the infusion start
T30: 30 min after the infusion start
NRS: Numerical Rating Scale
MMDs: Monthly migraine days
Cmax: Maximal serum concentration
MAMs: Number of monthly acute medications
MOH: Medication overuse headache
T60: 1 h after the infusion start
T90: 1.5 h after the infusion start
T120: 2 h after the infusion start
Fab: Fragment antigen-binding
mAb: Monoclonal antibody
SD: Standard deviations
IQR: Interquartile range
ORs: Odds ratios
CIs: Confidence intervals
MBS: Most bothersome symptoms
